# Influence of Preoperative Anxiety Level on Postoperative Pain After Cardiac Surgery

**DOI:** 10.7759/cureus.22170

**Published:** 2022-02-13

**Authors:** Muhammad Kashif, Mohammad Hamid, Amir Raza

**Affiliations:** 1 Department of Anesthesiology, Aga Khan University Hospital, Karachi, PAK

**Keywords:** clinical anxiety, preoperative period, postoperative period, cardiac surgical procedures, pain, anxiety

## Abstract

Background

Preoperative anxiety is generally neglected in the evaluation of cardiac surgery patients due to various reasons including insufficient literature and lack of simple assessment tools. In addition to this, the association between anxiety and postoperative complications including pain has been scarcely studied. The present study was designed to assess preoperative anxiety levels in all patients coming for cardiac surgery and then evaluate the effect of different levels of anxiety on postoperative pain scores.

Methods

This prospective cohort study was conducted in a single university hospital from March 2018 to December 2019. One hundred consecutive cardiac surgery patients between the ages of 18-65 years were enrolled in this study. The level of preoperative anxiety (assessed by State Anxiety Inventory) and its effect on postoperative pain and morphine consumption was assessed.

Results

The average age of the patients was 58.24±10.03 years of which 68% were male and 32% were female. Preoperative mild anxiety was observed in 64% of patients and moderate to severe anxiety in 36% of patients. Post-operative mean pain score was significantly high in the moderate to severe anxiety group as compared to the mild anxiety group [Mean pain difference =1.64 (95%CI: 1.38-1.89) p=0.0005], [Mean pain difference =0.51 (95%CI: 0.29-0.73) p=0.0005] at 12 hours and 24 hours respectively. Intraoperative and postoperative morphine consumption was significantly high in patients with moderate to severe anxiety.

Conclusions

Patients with moderate to severe anxiety before cardiac surgery experienced higher pain scores at a post-operative period which is significantly different from the mild anxiety group. Intraoperative and postoperative analgesic requirements were also significantly increased.

## Introduction

Anxiety is defined as a negative or life-threatening emotion that one feels generally in the ong-term or in a specific situation, that fluctuates over time [1]. Surgery and hospitalization are considered to be major life changes, causing anxiety regardless of the type of surgery and disease [2]. The degree to which each patient manifests anxiety depends on many factors including age, gender, type and extent of the proposed surgery, previous surgical experience, personal susceptibility to a stressful environment, and socioeconomic and ethnic background [3].

Heightened anxiety before surgery is associated with hypertension, dysrhythmias, surgery refusal, exacerbation of coronary artery disease symptoms [4], slow wound healing, fluid and electrolyte imbalance, and increased risk of infection. Heightened anxiety has also been linked to post-operative pain, impaired quality of life, and increased morbidity and mortality [5, 6]. Preoperative anxiety may have a role in the development of chronic postoperative pain, but more studies are needed [7, 8]. The reported incidence of preoperative anxiety ranges from 60-92% in unselected surgical groups and is higher in females [9, 10]. Nigussie et al. in 2012 found a significant rate of preoperative anxiety (70.3%) among surgical patients [2]. Prevalence of anxiety is probably higher in cardiac surgical patients while on the waiting list [11].

There are few studies that showed a significant correlation between preoperative anxiety and postoperative pain [12-15]. Approximately 30-80% of patients undergoing surgery suffer from inadequately treated pain, and preoperative anxiety has been seen to influence patients to experience more pain after surgery [16]. Miguel et al. in their study concluded that preoperative anxiety significantly increases postoperative pain and analgesic consumption in cardiac surgery patients [17].

The State Anxiety Inventory (STAI) for Adults is an excellent tool for assessing anxiety. The reliability and validity of STAI are well reported. The STAI form (Y) is the definitive instrument for measuring anxiety in adults. The STAI Form Y-6 consists of six statements that evaluate how the patient feels “right now” at this moment.

Pain is a subjective experience that is influenced by psychological, cultural, and other variables. The most common way to quantify pain is through self-reporting measures. A patient may describe pain intensity simply by indicating his pain on a scale. Most of these scales are unidirectional. These scales are simple and easy for the patients to use and understand and are relatively inexpensive.

The visual analog scale (VAS) and numerical rating scale (NRS) are commonly used scales to assess pain. Studies have shown a good correlation between the VAS and other measurements of pain intensity. In addition, VAS and NRS are easy to understand and are reproducible over time. The numerical rating scale is used to determine the intensity of pain. Patients would be asked to rate their pain from “0” (no pain) to 10 (pain as intense as it could be). This scale is reliable, reproducible and easy to administer. Additionally, it is readily acceptable to the patient.

Generally, there is an inadequate analysis of a patient’s psychological status pre-operatively, which hinders appropriate measures to reduce the patient’s anxiety. This issue needs to be addressed to reduce postoperative complications including pain.

Preoperative anxiety is generally neglected due to various reasons including lack of knowledge, insufficient literature, and lack of simple assessment tools. The results of this project may also be beneficial to the anesthesia community and societies, by potentially heightening the awareness about preoperative anxiety and its effects where this is not typically considered. This awareness may lead to better prevention and readiness through the creation or improvement of policies, procedures, and training.

The present study was designed to assess preoperative anxiety levels in all patients coming for cardiac surgery and then evaluate and compare with postoperative pain scores.

A preprint of this article was published in Research square.

## Materials and methods

This prospective cohort study was conducted in the preoperative area, ward & high dependency unit of Aga Khan University Hospital, Karachi. One hundred consecutive patients between the ages of 18-65 years were enrolled in this trial. All patients coming for cardiac surgery and planned for fast-track extubation were included. Emergency cardiac surgery patients and those who required reopening for bleeding were excluded from the study. Other excluded patients were patients taking any antipsychotic or anxiolytics, those suffering from Hypo- or hyperthyroidism, and neurological or psychological disorders.

The study was conducted after approval from the institutional ethical review committee (ERC). The duration of the study was between March 2018 to December 2019. Informed written consent was taken the night before surgery from all the patients scheduled to undergo elective cardiac surgery. A copy of the informed consent was given to the patient. The confidentiality of the patient and data was maintained by assigning a number for each patient data. Electronic data was password protected and data on hard copies were kept in a lock and key.

Non-probability consecutive sampling technique was used. Those patients who fulfilled the inclusion criteria were approached a night before surgery. Patients were assessed pre-operatively by using the State Anxiety Inventory (STAI) scale for adults. The STAI Form Y-6 consists of six statements that evaluate how the patient feels “right now” at this moment. The primary investigator asked these questions and answers were noted in a proforma and scoring was done. Individuals with an STAI score of more than 40 were considered as moderate to severe anxiety, and those having an STAI score of 40 or less were allocated mild anxiety group. These patients were approached after successful surgery and extubation. Anxiolytics were not given postoperatively. All patients received intravenous Tramadol 50 mg 8 hourly and intravenous paracetamol 1 gram 6 hourly, while 1 mg of intravenous morphine was given on PRN basis. The post-operative pain was determined by using a numeric pain scale at 12hr and 24hrs of surgery and total narcotic consumption was also noted along with the duration of surgery, amount of intraoperative and postoperative analgesia used. Pre-operative anxiety and post-operative pain assessment were done by the primary investigator on a predesigned data collection form.

Sample size calculation was based on a previous study by Koivula et al. who reported the preoperative anxiety as 50% in coronary artery bypass graft surgery (CABG) [11]. One hundred patients were needed to estimate the expected prevalence of preoperative anxiety within a 10% margin of error with a 95% confidence interval. 

Data were analyzed by the statistical packages for social science version 17 (SPSS Inc., Chicago, IL). Mean and standard deviation were estimated for quantitative variables like age, weight, height, BMI and STAI scores, duration of surgery, intraoperative analgesics used, postoperative opioid consumption, and post-operative pain scores. The normality of the continuous data was tested by the Shapiro-Wilk test and by examining the quantile plot. Normally distributed continuous variables were presented as mean ± SD and compared between the mild vs moderate to severe anxiety using a two-sample Student t-test. When the distribution was not normal, the median along with the first (Q25) and third quartiles (Q75) was presented, and a Mann-Whitney U test was used. Frequencies and percentages were calculated for qualitative variables like gender, marital status, and type of surgery and analyzed by chi-square or Fisher’s exact test. The general linear model was used to observe the association of preoperative anxiety and pain score (continuous dependent variable) after controlling the effect of age, gender, type of surgery, diabetes, and hypertension. Regression coefficients were reported for each independent variable. A P-value ≤0.05 was considered significant.

## Results

A total of 100 patients undergoing cardiac surgery (CABG or valvular surgery) were included in the study. The average age of the patients was 58.24±10.03 years of which 68% were male and 32% were female. Out of 100 patients, CABG was performed in 88% and the rest were 12% (valvular surgery). Diabetes mellitus was observed in 55% and 70% were hypertensive.

Out of 100 patients, preoperative mild anxiety was observed in 64% of patients and moderate to severe anxiety in 36% of patients. Demographic characteristics, type of surgery, and duration of surgery were not statistically significant in patients with mild anxiety and the moderate to severe anxiety groups (Table 1).

**Table 1:** Demographic and other characteristics of patients (n=100)

Variables	Mild Anxiety n=64	Moderate to severe Anxiety n=36	p-value
Age (years)	59.23±9.75	56.47±10.42	0.18
Weight (kg)	71.04±12.63	65.56±12.36	0.03
Height (cm)	162.11±10.57	160.51±9.87	0.46
BMI (kg/m^2^)	27.27±4.98	26.24±4.15	0.31
Male / Female	45(70.3%) / 19(29.7%)	23(63.9%) / 13(36.1%)	0.51
Smoker	26(40.6%)	12(33.3%)	0.47
Hypertension	43(67.2%)	27(75%)	0.41
Diabetes Mellitus	31(48.4%)	24(6.7%)	0.08
Others Comorbids	13(20.3%)	5(13.9%)	0.42
Valvular heart surgery	7(10.9%)	5(13.9%)	0.66
CABG	57(89.1%)	31(86.1%)	

The postoperative mean pain score was significantly high in the pre-operative moderate to severe anxiety group as compared to the mild anxiety group [Mean pain difference =1.64 (95%CI: 1.38-1.89) p=0.0005], [Mean pain difference =0.51 (95%CI: 0.29-0.73) p=0.0005] at 12 hours and 24 hours respectively. Intraoperative and postoperative morphine consumption was significantly high in patients with moderate to severe anxiety groups (Table 2).

**Table 2:** Comparison of postoperative pain score and intra and postoperative analgesia in patients with mild and moderate to severe anxiety

Outcome	Mild Anxiety n=64	Moderate to severe Anxiety n=36	P-value
Pain at 12 hours	3.50±0.67	5.14±0.54	0.0005
Pain at 24 hours	1.38±0.49	1.89±0.57	0.0005
Intra-operative Analgesia morphine (mg)	12.78± 1.22	13.81±1.72	0.001
Post-operative analgesia Morphine (mg)	3.84±4.59	11.39±6.93	0.0005

Multivariate analysis at 12 hours, the adjusted beta coefficient of preoperative moderate to severe anxiety was 1.71 [95%CI: 1.44 to 1.98] which demonstrated that on average postoperative pain score was significantly higher in patients with preoperative moderate to severe anxiety than those who had preoperative mild anxiety after controlling the age, gender, BMI, type of surgery and comorbid like diabetic and hypertension (Table 3).

**Table 3:** Multivariable analysis by using General Linear Model showing the association of preoperative anxiety and pain score Pain score at 12 and 24 hours are dependent variables.  Adjusted R Square = 0.626 and 0.28 for 12 hours and 24 hours respectively.    Β = Regression Coefficient; Se= Standard Error ; * Significant p-value

Predictors	Pain at 12 hours			Pain at 24 hours		
	Β(SE)	95%CI	P-value	Β(SE)	95%CI	P-value
Mild Anxiety	Ref			Ref		
Moderate to severe Anxiety	1.71(0.14)	1.44 to 1.98	0.0005*	0.52(0.11)	0.29 to 0.74	0.0005*
Age (Years)	-0.006(0.007)	-0.02 to 0.07	0.363	-0.002(0.01)	-0.013 to 0.009	0.75
Female	Ref			Ref		
Male	0.09(0.14)	-0.18 to 0.36	0.518	0.17(0.11)	-0.05 to 0.40	0.12
BMI	0.007(0.01)	-0.02 to 0.03	0.519	0.002(0.01)	-0.02 to -0.017	0.841
Valvular surgery	Ref			Ref		
CABG	0.11(0.23)	-0.36 to 0.58	0.634	-0.47(0.19)	-0.86 to -0.09	0.016*
Diabetic Mellitus	-0.31(0.14)	-0.59 to -0.03	0.032*	-0.08(0.12)	-0.31 to 0.16	0.51
Hypertension	-0.13(0.15)	-0.43 to 0.17	0.412	0.02(0.13)	-0.23 to 0.27	0.86

Similarly, at 24 hours, the magnitude of the adjusted beta coefficient of preoperative moderate to severe anxiety was lower than 12 hours but still statistically significant.

## Discussion

Anxiety is a common disorder that involves feelings of extreme fear or worry. It has been observed that many patients undergoing surgery experience anxiety. It has a positive relationship with intraoperative and postoperative complications. There are various tools designed for measuring anxiety and all those tools have some limitations. Among them, the STAI questionnaire is considered the most reliable tool as it has shown consistent results in various studies and is a sensitive predictor of distress over time.

The incidence of significant preoperative anxiety in our study population was 36% using the STAI scale. Miguel et al. also found the incidence [5] of anxiety to be 32% while in other studies it varies from 20-92% [2, 9, 18]. This wide range may be due to the use of different scales in different surgical patients. The type of population may also influence the incidence of anxiety as higher percentages are seen in a western population [11, 19].

Determinants of high preoperative anxiety in various studies are prolonged waiting time, age under 65 years [5], fear of anesthesia and surgery, lack of information about the procedure, fear of postoperative pain and distorted body image, and separation from family. Better preoperative consultation has been shown to reduce anxiety [20, 21].

In the present study, although insignificant, preoperative anxiety was more common in female cardiac surgery patients than male patients (40.6% vs 33.8%). This is in line with the study by Mehdi et al. who suggested that the preoperative anxiety levels are much higher in female than male patients [1]. Vilma et al. also found higher anxiety in females [22]. Although, the percentage of female patients was very low in the present study and difficult to conclude, previous studies have also shown the same trend [9].

No significant differences were found between preoperative anxiety and smoking, diabetes mellitus (DM), hypertension (HTN), and BMI. Type of surgery (valvular vs CABG) also had no significant effect on anxiety. Valve surgery patients were slightly more anxious preoperatively in comparison with CABG surgery patients. This is in contrast with other studies where CABG patients showed higher anxiety scores [23]. The reason may be that in our study only 12 patients underwent valve surgery and the rest were CABG surgery patients.

A study by Kil et al in 2011 concluded that patients with higher STAI scores required a greater amount of induction and inhalational agent and were also significantly correlated with postoperative pain [24]. We did not examine induction agent and inhalation requirements, but intraoperative analgesia requirements were increased in the moderate to severe anxiety group. Rather, intraoperative analgesia requirements in the present study were significantly higher in the moderate to severe anxiety group. Shiho et al. proposed that high preoperative anxiety is associated with reduced intraoperative nociceptive response (changes in HR, SBP, and perfusion index) and high postoperative response [25]. This may be the reason that intraoperative opiate consumption was lower than expected in their study. Preoperative anxiety may also affect intraoperative hemodynamics. Bayrak et al. noticed a higher heart rate and blood pressure in the anxiety group than non-anxiety or lower anxiety score groups [26]. Post-operative pain scores were also higher in the anxiety group. Marentes et al. demonstrated higher anesthetic requirements in the anxiety group but analgesic requirements were not mentioned in their study [27]. In fact, we were unable to find any study showing intraoperative analgesia requirements. We need further studies to look at this factor as well.

In our study, using the standard analgesia protocol, postoperative pain scores were significantly higher in the moderate to severe anxiety group as compared to the mild anxiety group in the first 24 hrs. As expected, the analgesic requirements were also significantly increased in the moderate to severe anxiety group. This may be due to the fact that the average pain score was moderate in the anxiety group and mild in non-anxiety during the first 12 hrs. A highly significant association was found when postoperative pain was compared between non-anxious and anxious patients after adjusting age gender, type of surgery, and other comorbidities. Our study is in line with a study by Miguel et al. who measured preoperative anxiety and depression using the hospital anxiety and depression scale (HADS) and found a significant correlation of pre-operative anxiety with postoperative pain [5]. Analgesic consumption was also high in their study in the anxiety group and remain high for 48 hours after extubation.

A study by Gresztat et al. in 2008 concluded that patients with higher preoperative anxiety respond poorly to analgesic medication, which may be due to increased perception of pain [28]. It is difficult to say whether preoperative anxiety leads to a change in pain intensity or if the response to pain medications is disturbed.

Ocalan et al. looked at ENT surgeries and found a positive relationship between preoperative anxiety and postoperative pain, but a negative relationship between postoperative pain with depression. They also suggested early intervention to relieve preoperative anxiety [29]. Other studies also concluded with the same suggestions in various surgeries [18, 30].

Generally, female patients have higher pain scores than men. In the present study, male patients showed slightly higher mean postoperative pain scores irrespective of groups during the first 24 hours as compared to females. However, the association between gender and postoperative pain was insignificant. The postoperative morphine requirement was higher in female patients than males but this association was insignificant.

Pregabalin has been used as an anxiolytic agent before neurosurgery and we were able to demonstrate the anxiolytic effect and reduced postoperative analgesic requirements. It shows that anxiolysis does reduce postoperative analgesic needs.

A significant association was also found between the type of surgery and postoperative pain at 24 hours, whereas postoperative pain was more at valvular surgery than CABG after adjusting other factors.

Further studies are required to see the effect of preoperative anti-anxiety medication on postoperative pain management. These medications can be started on surgical floors the night before surgery and continued till the patient is called to the operative room. In addition, it is the responsibility of doctors and nurses to educate patients about the procedure and also take appropriate interventions in order to reduce anxiety and postoperative morbidities. Another approach is to give ICU tours to patients and families before surgery in order to improve satisfaction and reduce anxiety.

The average age of the anxiety group was non-significantly lower than the non-anxiety group. However, postoperative pain was not affected by age, except in the 51 to 60 years group. There was no change in analgesic requirements for pain control in both groups despite higher pain scores in the anxiety group.

Our study also found that patients of the anxiety group having a duration of surgery of more than 300 minutes had the greatest post-operative pain scores (5.00 ± 1.41) at 12 hours. which is moderate by our definition. This is significantly different for non-anxious patients who had mild pain at the same time. However, this difference resolved at 24 hrs.

The main limitation of the study was that the study was performed in only one center and all factors responsible for preoperative anxiety, like waiting time and previous surgery experience, were not considered. Other outcomes like delirium, length of stay, and quality of life after surgery were also not reported.

## Conclusions

In conclusion, our study indicates that the patients undergoing cardiac surgery and those who experienced moderate to severe anxiety before surgery are more prone to develop higher pain scores in the postoperative period which is significantly different from the mild anxiety group. Intraoperative and postoperative analgesic requirements were also significantly increased in moderate to severe preoperative anxiety patients.
